# Tibial Tubercle Osteotomy With Anteriorization and Distalization for Treatment of Patellar Instability With Patella Alta

**DOI:** 10.1016/j.eats.2022.02.009

**Published:** 2022-05-17

**Authors:** Joseph Temperato, Clayton W. Nuelle

**Affiliations:** a Department of Orthopaedic Surgery, University of Missouri, Columbia, Missouri, U.S.A.; b Thompson Laboratory for Regenerative Orthopaedics, University of Missouri, Columbia, Missouri, U.S.A.

## Abstract

Patellofemoral instability is a common cause of knee pain that can lead to long-standing pain, chondral injury, recurrent dislocations, and degenerative changes if not treated appropriately. Tibial tubercle osteotomy is indicated when there is anatomy predisposing to patellar maltracking and instability, namely abnormal patellar height or tibial tubercle location. In this Technical Note, we describe a technique for tibial tubercle anteriorization and distalization as part of the overall treatment algorithm for patellar instability with associated patella alta. This method of tibial tubercle osteotomy reliably produces anterior and distal translation of the patella to correct patellar height and decrease contact pressure across the patellofemoral joint.

Patellofemoral instability is a common cause of patellofemoral pain, affecting between 7 and 49 persons per 100,000.^1^ There is a spectrum of patellar instability, which can range from subluxation to frank dislocation. Patients who sustain a first-time patellar dislocation are most commonly female individuals aged between 10 and 19 years and involved in athletic activity.^2^^,^^3^ Additionally, there are several anatomic risk factors that predispose to patellar instability, including trochlear dysplasia, patella alta, elevated tibial tubercle–trochlear groove distance, and increased lateral patellar tilt.^4^ In particular, patella alta is present in 24% of individuals with patellar instability but only 3% of normal controls.^5^ It has been shown to contribute to greater lateral displacement, greater lateral tilt, and less contact area^6^ and to be a contributor to lateral patellar facet chondral degeneration.^7^

Most patients with a single dislocation event can be treated nonoperatively in the absence of a loose body or osteochondral injury.^8^ Nonoperative treatment consists of a brief period of immobilization followed by physical therapy, which has traditionally focused on restoration of range of motion and quadriceps strengthening.^9^ In current practice, it is also recommended that physical therapy focus on strengthening of the hip abductors and external rotators as well.^10^ However, the risk of redislocation is between 22% and 37% after a first-time dislocation, which is not negligible.^11^^,^^12^ This high redislocation rate has led to some advocacy for early surgical management of patellar instability to lower the probability of subsequent dislocation.^13^

Although there is some debate about the optimal treatment strategy, a thorough preoperative workup is essential to a successful outcome because unaddressed bony malalignment can lead to failure of isolated soft-tissue procedures.^14^ In the treatment of instability, procedures that address patella alta reduce the incidence of recurrent dislocation and improve outcomes.^15^, ^16^, ^17^, ^18^ They also decrease contact pressures across the patellofemoral joint.^19^ A number of osteotomy techniques have been described, with distalization of the tubercle being the primary technique to correct patella alta.^20^, ^21^, ^22^ In this Technical Note, we describe a technique for tibial tubercle anteriorization and distalization as part of the treatment for patellar instability with associated patella alta.

## Surgical Technique

### Patient Evaluation and Imaging

**Fig 1:**
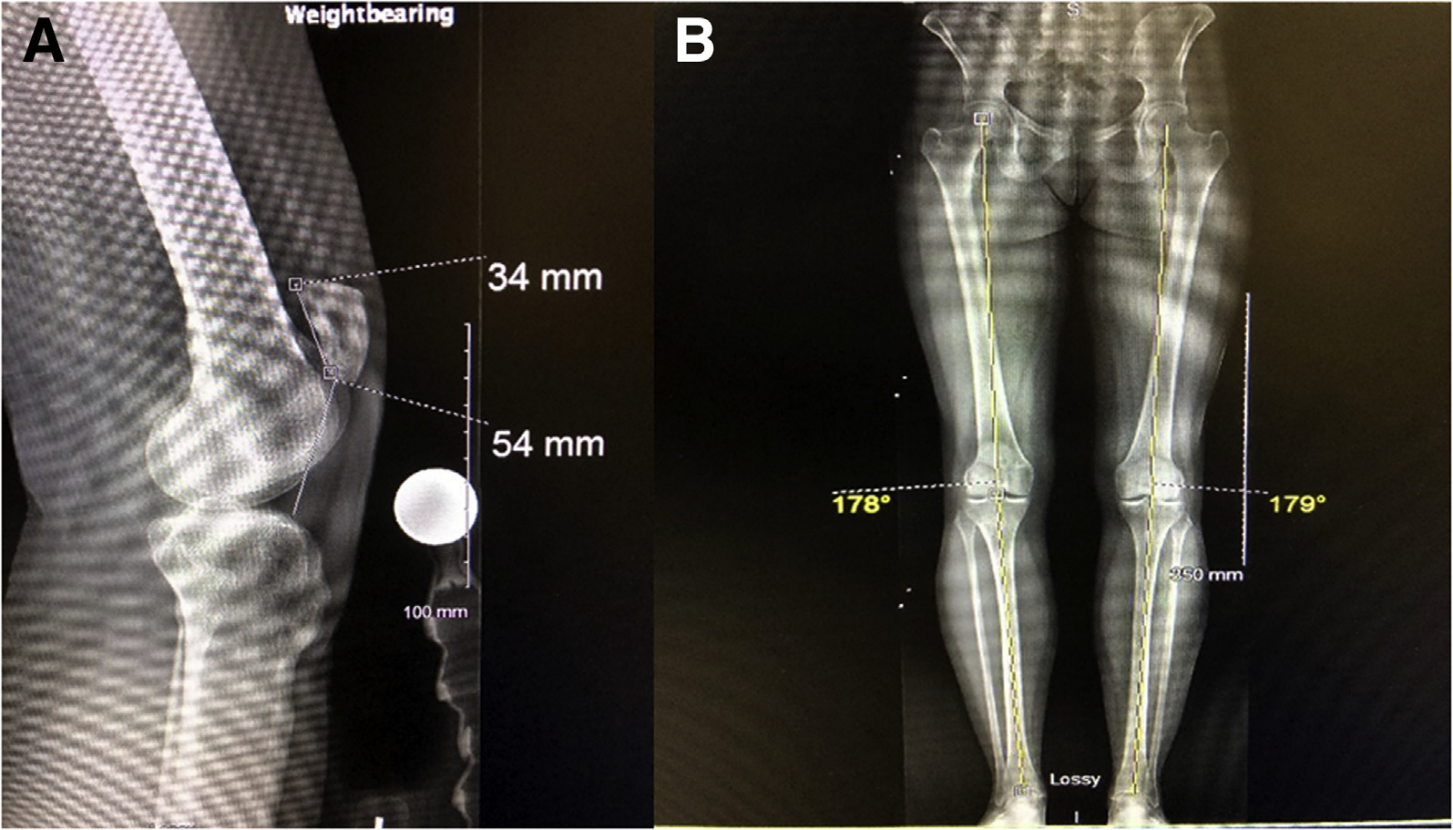
Preoperative radiographs prior to left knee tibial tubercle osteotomy. (A) A lateral radiograph shows patella alta with a Caton-Deschamps index of 1.59. (B) A standing lower-extremity alignment radiograph shows no coronal-plane malalignment.

The initial patient evaluation should consist of a thorough history including a description of symptoms, history of trauma, and any procedures previously performed on the knee in question. The imaging workup consists of weight-bearing radiographs of the knee (Fig 1A) as well as standing alignment radiographs of the bilateral lower extremities (Fig 1B) to evaluate for malalignment. A preoperative magnetic resonance imaging scan of the knee may also be ordered to evaluate for chondral pathology. This may be helpful in determining whether additional procedures are indicated in addition to tibial tubercle distalization. During preoperative planning, it is essential to determine whether additional procedures will be performed at the time of distalization or in a staged fashion.

The patient is positioned supine on the operating table by use of a basic knee arthroscopy setup per surgeon preference. Examination of the patient under anesthesia is performed, including evaluation of lateral quadrant translation of the patella when medial-to-lateral pressure is applied. This should be performed throughout the arc of motion from full extension to deep flexion.

Standard arthroscopic portals are made, and a thorough diagnostic arthroscopy is performed to evaluate and address any concomitant pathology. Careful evaluation of the patellofemoral joint is performed because chondral damage commonly occurs here in patients with malalignment. A chondroplasty is performed with an oscillating shaver as needed. The arthroscope is withdrawn, and attention is turned toward the open part of the procedure.

**Fig 2:**
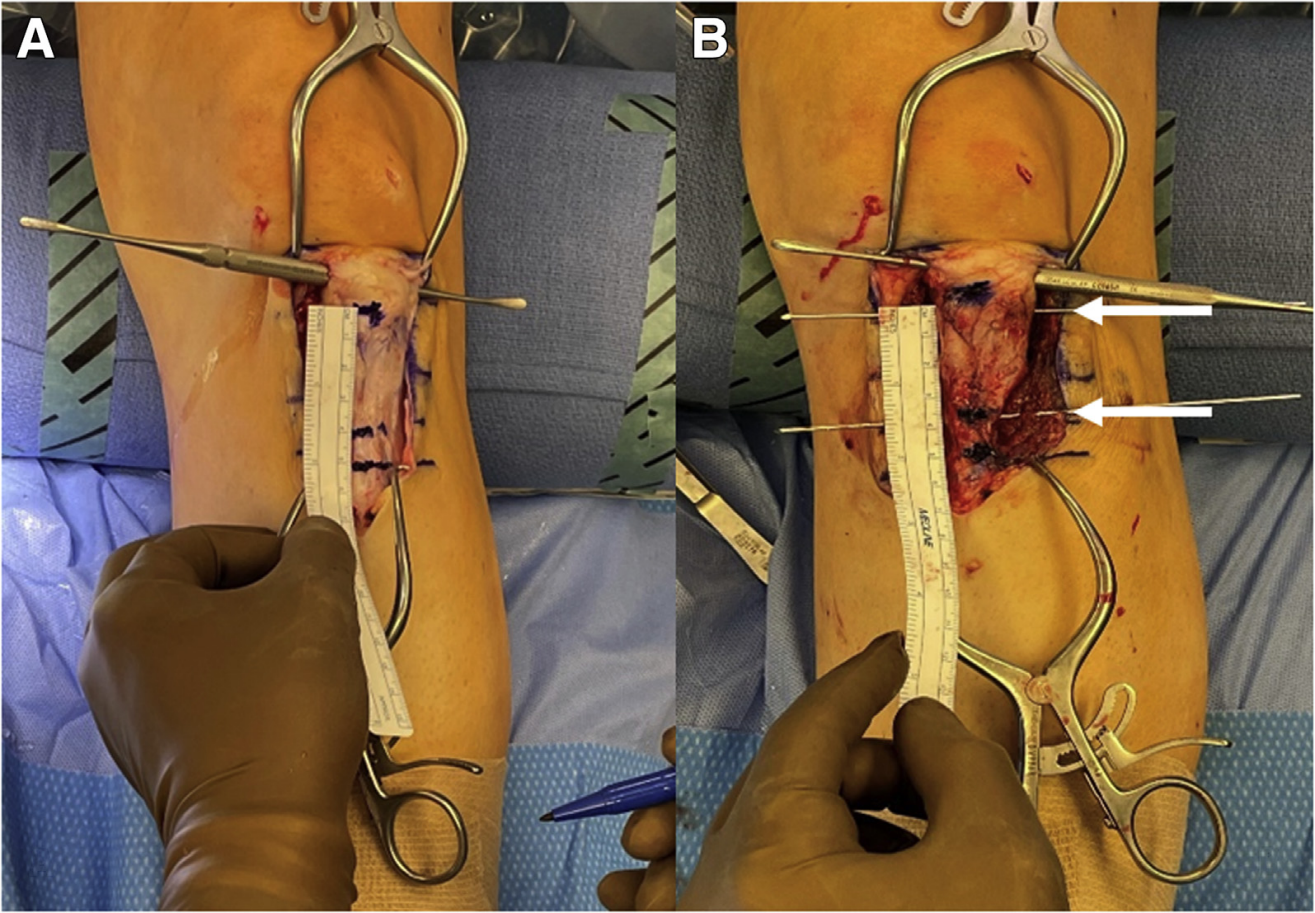
Intraoperative images of a left knee (where the top of the images is cranial) showing tibial tubercle measurements prior to distalization tubercle osteotomy. (A) The proximal and distal aspects of the tibial tubercle are marked and measured with a sterile ruler to compare with measurements made on preoperative imaging. (B) Two K-wires (white arrows) are placed parallel to one another at the proximal- and distal-most aspects of the tibial tubercle at a depth of approximately 8 to 9 mm.

A No. 10 blade scalpel is used to make a midline incision over the tibial tubercle. Skin flaps are elevated using a combination of a scalpel, Bovie electrocautery (Bovie Medical, Clearwater, FL), and blunt dissection. Once adequate exposure is achieved, a ruler is used to measure the length of the patellar tendon, where it inserts at the proximal-most aspect of the tibial tubercle, as well as its distal-most insertion on the tibial tubercle, as shown in Fig 2. Two K-wires are then placed parallel to one another in a medial-to-lateral fashion at the proximal- and distal-most aspects of the tibial tubercle at a depth of approximately 8 to 9 mm (Fig 2). A third mark is made, using the precalculated distance by which the tubercle will be distalized, distal to the distal aspect of the tibial tubercle (Fig 2). This serves as a marker of bone to be resected to allow for tubercle distalization.

**Fig 3:**
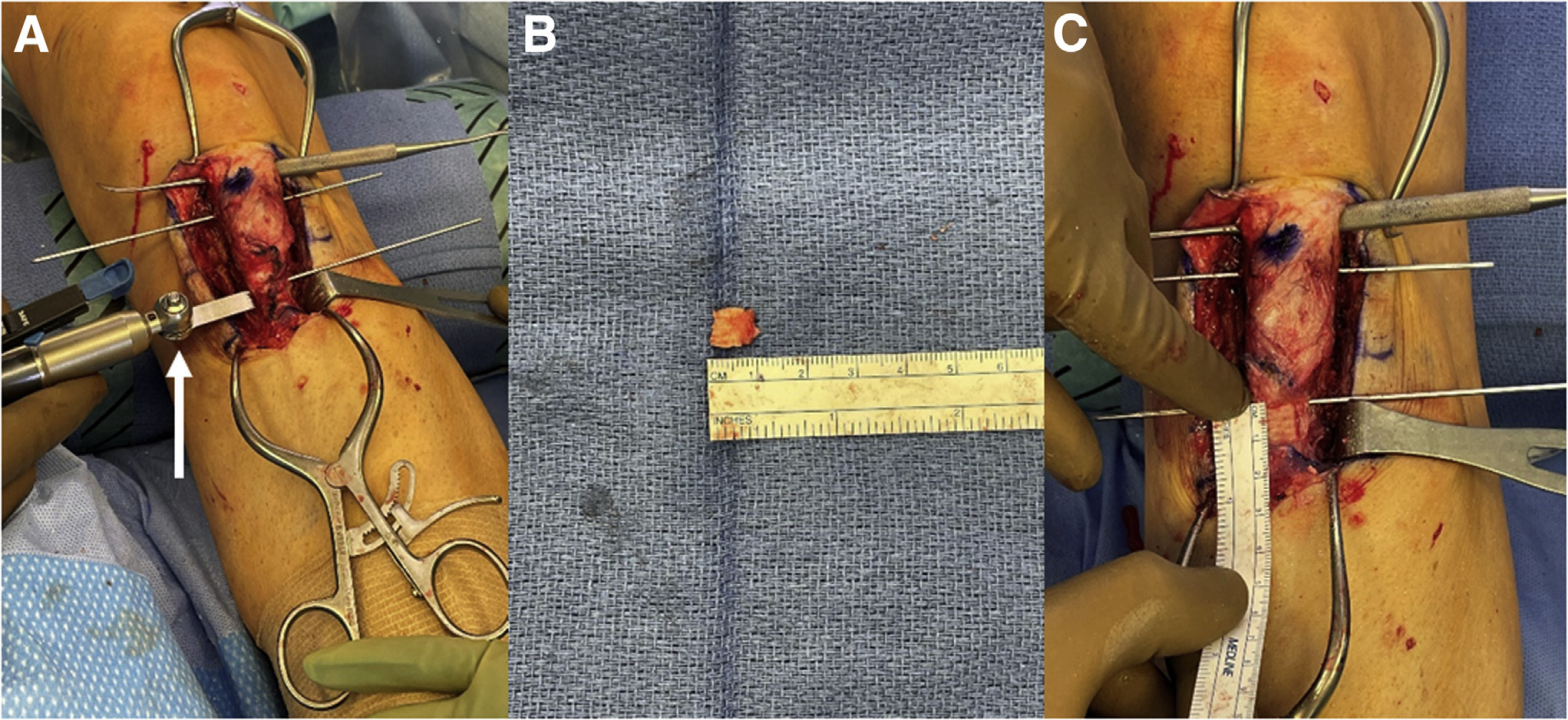
Intraoperative images of a left knee showing steps of distalization tibial tubercle osteotomy. (A) A TPS saw (white arrow) is used to perform measured bone block resection distal to the osteotomy shingle. (B, C) After the bone block is removed, its size is confirmed on the back table (B) and at the resection site (C).

By use of the aforementioned K-wires as a reference, a sagittal saw is used to perform an osteotomy of the tibial tubercle in a medial-to-lateral fashion with a slope of approximately 10° to 15°. A 10-mm-wide TPS saw (Stryker, Kalamazoo, MI) is then used to make an anterior-to-posterior cut at the third mark distal to the tubercle osteotomy site at a depth of 5 to 6 mm. Osteotomy of this distal cortical resection fragment is completed in a medial-to-lateral fashion, and the cortical fragment is removed (Fig 3). Osteotomy of the proximal tubercle shingle is then completed with an osteotome.

**Fig 4:**
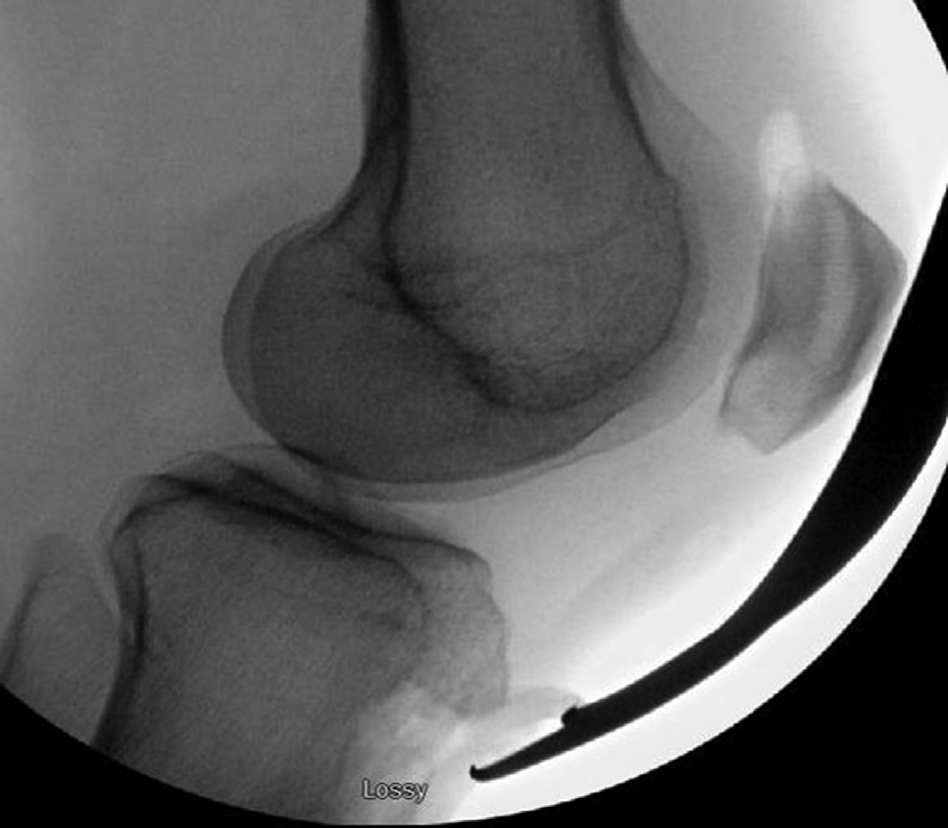
Intraoperative lateral X-ray fluoroscopic image of a left knee after tibial tubercle distalization osteotomy to confirm that the tubercle is appropriately positioned and patellar height is appropriate.

**Fig 5:**
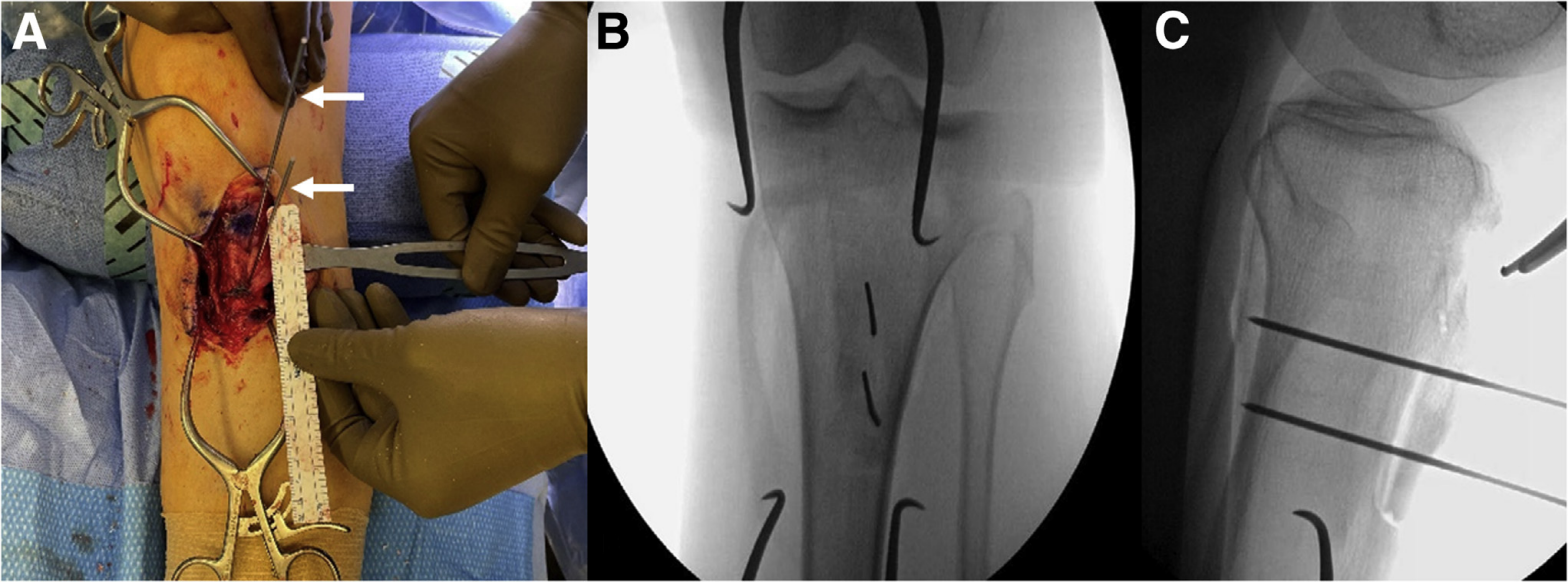
Intraoperative images of a left knee showing fixation technique for tibial tubercle after distalization osteotomy. (A) Two bicortical K-wires are placed in an anterior-to-posterior direction approximately 15 mm apart and equidistant from the osteotomy shingle ends (white arrows). (B, C) Tubercle and K-wire positioning is again confirmed with intraoperative fluoroscopy on anteroposterior (B) and lateral (C) views.

**Fig 6:**
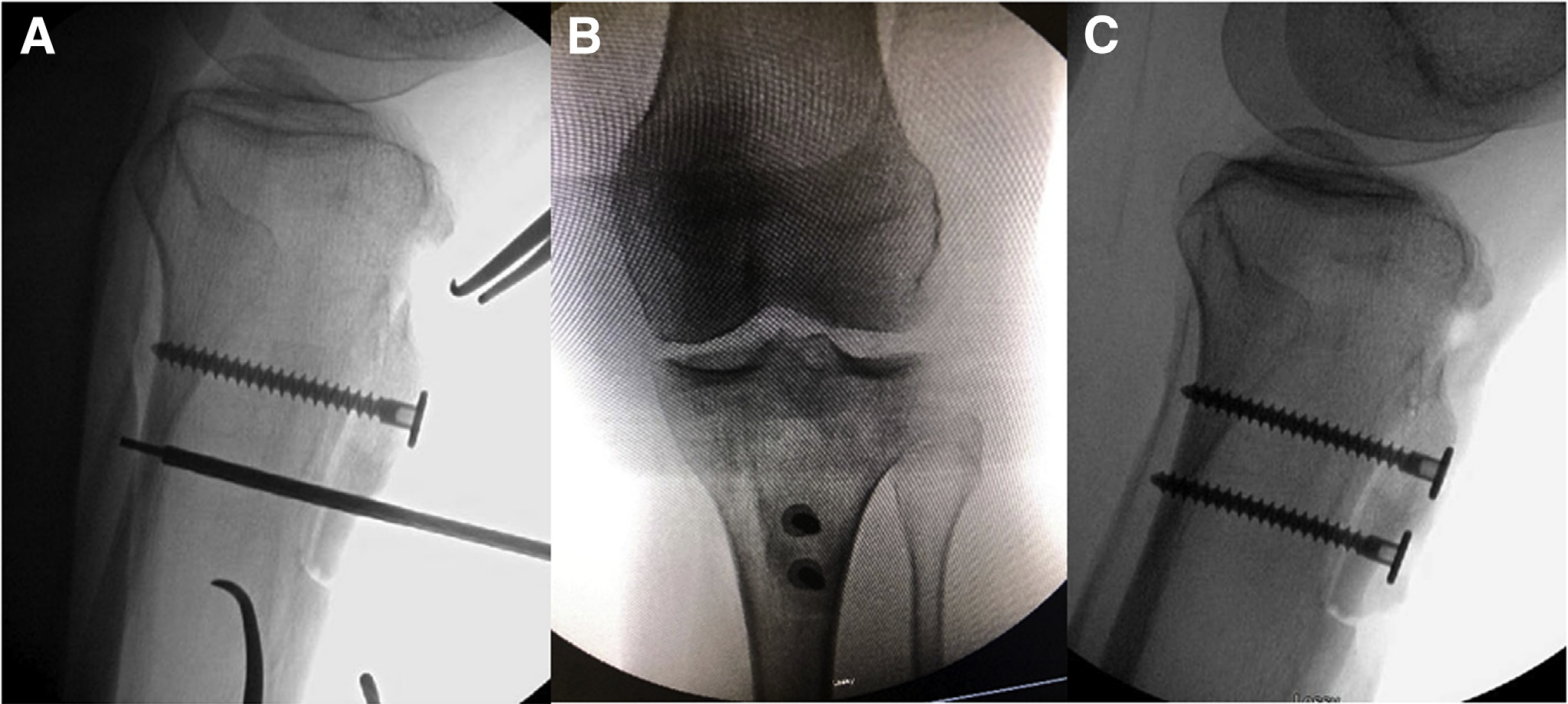
Intraoperative images of X-ray fluoroscopy of a left knee showing tibial tubercle fixation after distalization osteotomy. (A) Overdrilling of the proximal cortices is performed, followed by placement of 2 fully threaded 4.5-mm screws (Arthrex) in an anterior-to-posterior fashion. (B, C) Fluoroscopy is used to again confirm final screw positioning on anteroposterior (B) and lateral (C) views.

Next, the tibial tubercle is distalized to the precalculated amount by moving the shingle to the distal extent of the area of resected cortical bone. Intraoperative fluoroscopy is used to confirm that the tubercle is distalized and anteriorized (Fig 4) to restore the appropriate patellar position. Two bicortical K-wires are then placed in an anterior-to-posterior direction approximately 15 mm apart and equidistant from the osteotomy shingle ends (Fig 5). Tubercle and K-wire positioning is again confirmed with fluoroscopy. The proximal cortices are then over-drilled, and 2 fully threaded 4.5-mm screws (Arthrex, Naples, FL) are inserted in an anterior-to-posterior fashion (Fig 6).

**Fig 7:**
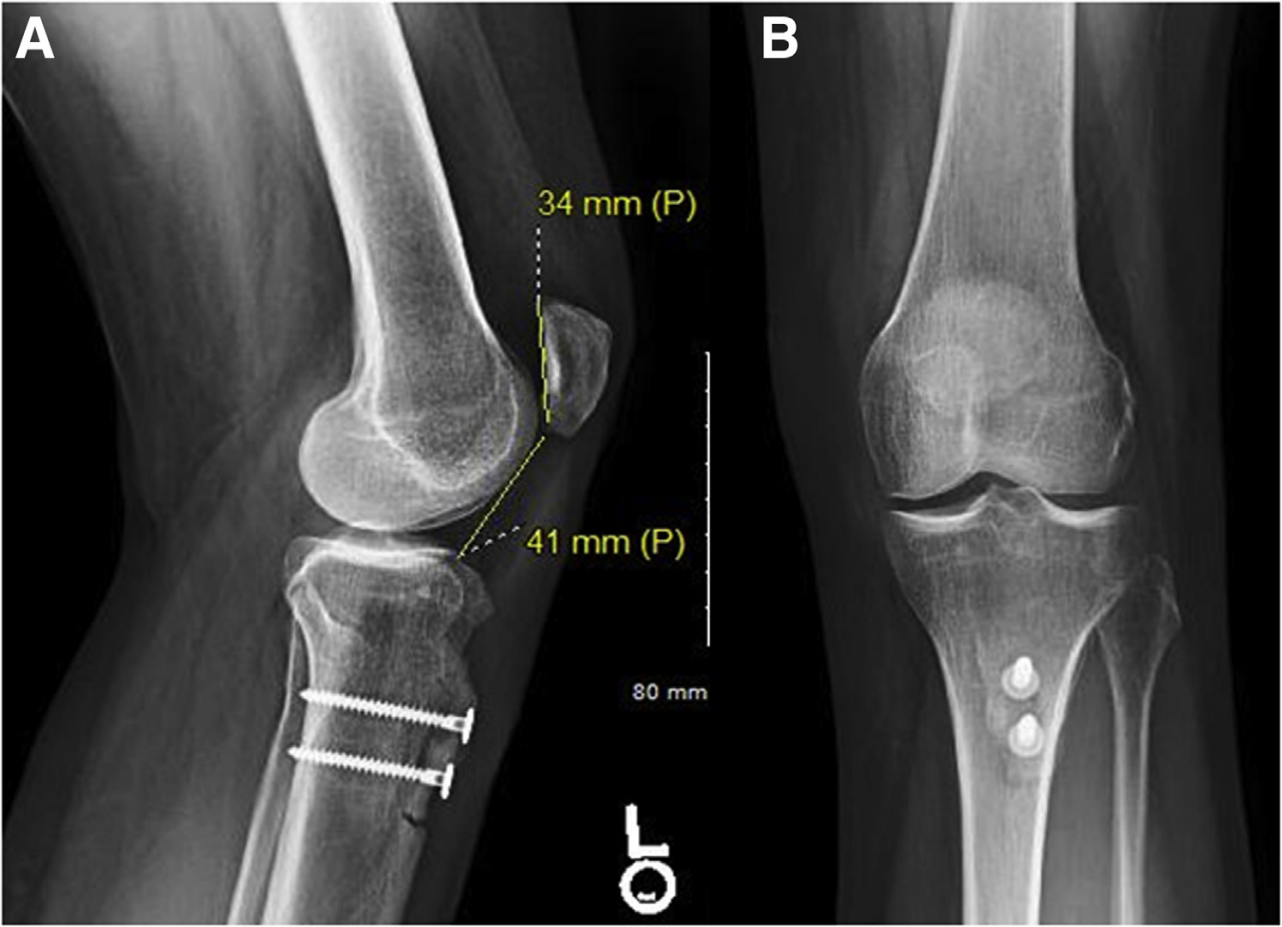
Postoperative radiographs of a left knee after tibial tubercle distalization osteotomy. (A) Lateral radiograph showing improved patellar height with Caton-Deschamps index of 1.2. (B) Anteroposterior radiograph showing final screw location and osteotomy position.

After final confirmation of screw placement and tubercle position on fluoroscopic anteroposterior and lateral views, the wound is irrigated and closed in a layered fashion. A soft dressing is placed, followed by a hinged knee brace locked in extension. A postoperative radiograph showing a knee with an improved Caton-Deschamps ratio is shown in Fig 7. The aforementioned technique is demonstrated in Video 1.

### Rehabilitation

The patient is placed in a hinged knee brace at the conclusion of the procedure, which is locked in extension for the first 2 weeks postoperatively. The patient is restricted to toe-touch weight bearing for 6 weeks to protect osteotomy-site healing, followed by a progressive weight-bearing protocol thereafter. Initial range-of-motion restrictions consist of passive motion from only 0° to 30° for the first 2 weeks. Range-of-motion exercises are initiated at the 2-week postoperative visit, and motion is advanced 10° to 15° per week thereafter.

## Discussion

Patellofemoral instability is a common cause of knee pain that can lead to a number of painful sequelae, including chondral injury, recurrent dislocations, and degenerative changes if not treated appropriately. Surgical intervention is a treatment option for patients with a loose body present after a dislocation event or recurrent instability despite conservative treatment measures. A number of soft-tissue and bony procedures are used to address recurrent instability, including medial patellofemoral ligament repair or reconstruction, lateral lengthening, tibial tubercle osteotomy, and trochleoplasty. These procedures may be performed in isolation or in combination. Additionally, several cartilage restoration procedures are available to address any chondral injury from the dislocation. They may also be performed in a single operation or in a staged fashion. The decision is based on each patient’s anatomic considerations, as well as any associated pathology. Although all of these procedures address different aspects of patellar instability, tibial tubercle osteotomy is a reliable procedure to correct patella alta and a lateralized tubercle.

There are several advantages to the described technique (Table 1). First, this method of tibial tubercle distalization allows for correction of patellar height in patients with patella alta. Precise bone block resection allows for calculated distalization based on preoperative imaging. Measurements are also confirmed intraoperatively. Another advantage is that the osteotomy may be performed in multiple planes, including anteriorization of the tubercle, which offloads the patellofemoral joint. Anteriorization effectively decreases contact pressures between the patella and the trochlea, which may decrease the risk of further chondral injury. This is an important consideration not only for the existing native cartilage but also when performing associated cartilage restoration procedures.

**Table 1:** Advantages and Disadvantages of Tibial Tubercle Anteriorization and Distalization

Advantages
Distalization of the tibial tubercle improves patellar height and decreases patella alta.
Bone block resection allows for precise distalization.
Placing multiple bicortical screws provides a strong fixation construct for the osteotomy shingle.
The osteotomy may be performed in multiple planes, including anteriorization of the tubercle, which offloads the patellofemoral joint and decreases contact pressures.
Disadvantages
Distalization osteotomy can be technically demanding and requires meticulous preoperative planning and measurement.
Bone block resection could lead to a risk of postoperative fracture.
Revision osteotomy may require a period of protected weight bearing and a prolonged rehabilitation course postoperatively.
If concurrent procedures are planned for concomitant pathology, staged procedures may be necessary.

The described technique has some disadvantages and limitations (Table 1). Distalization osteotomy can be technically demanding and requires meticulous preoperative planning and intraoperative measurements. In addition, bone block resection requires removal of cortical bone, which could lead to the potential risk of postoperative fracture. In cases of revision osteotomy, in particular, a longer period of protected weight bearing and a prolonged rehabilitation course may be required postoperatively. Finally, if additional procedures are planned for concomitant pathology, staged procedures may be necessary. Despite these potential drawbacks, this technique for tibial tubercle osteotomy can reliably correct patella alta when treating patellar instability. Additional pearls and pitfalls to consider when performing this technique are shown in Table 2.

**Table 2:** Pearls and Pitfalls of Tibial Tubercle Anteriorization and Distalization

Pearls	Pitfalls
The surgeon should determine all pathology responsible for instability preoperatively to decide when additional procedures may be indicated. The surgeon should determine whether the procedures will be performed in a single operation or a staged fashion.	Failure to address all sources of instability may result in persistent instability and/or poor outcomes.
Preoperative imaging should be used to plan the appropriate amount of distal bone resection.	Imaging should include AP, lateral, sunrise, and full-length extremity alignment films to fully assess the patellofemoral alignment and overall limb alignment.
Preoperatively planned distalization and bony resection should be confirmed based on the intraoperative patellar tendon and tibial tubercle lengths.	
The surgeon should confirm placement of the planned osteotomy marked with K-wires using fluoroscopy in the AP and lateral planes prior to performing the osteotomy.	This particular technique requires freehand placement of K-wires for the planned osteotomy.
Use of a small TPS saw for distal bone block removal allows for a more precise osteotomy.	Use of a TPS saw blade requires a meticulous freehand technique and multiple instruments.
The surgeon should ensure adequate length and depth of the tubercle shingle to avoid potential screw cutout, fracture, or loss of fixation.	A shingle that is too thin may not hold fixation adequately or may result in postoperative fracture.
A minimum of 2-3 bicortical screws should be used for osteotomy shingle fixation.	Use of non-headless screws may result in screw head prominence and may require removal of hardware at a later time.

AP, anteroposterior.

## Supplementary Data

ICMJE author disclosure forms

Video 1 Technique to perform tibial tubercle osteotomy with anteriorization and distalization. Our surgical technique for the treatment of patella alta is described in a patient with patellar instability. Sagittal and TPS saws are used to make an angled osteotomy, and the bone shingle is secured with two 4.5-mm cortical screws after appropriate distalization to reduce patella alta.
